# Antioxidant Effect of Beer Polyphenols and Their Bioavailability in Dental-Derived Stem Cells (D-dSCs) and Human Intestinal Epithelial Lines (Caco-2) Cells

**DOI:** 10.1155/2020/8835813

**Published:** 2020-10-10

**Authors:** Marina Di Domenico, Antonia Feola, Pasqualina Ambrosio, Federica Pinto, Giovanni Galasso, Armando Zarrelli, Giovanni Di Fabio, Marina Porcelli, Salvatore Scacco, Francesco Inchingolo, Lucio Quagliuolo, Andrea Ballini, Mariarosaria Boccellino

**Affiliations:** ^1^Department of Precision Medicine, University of Campania “Luigi Vanvitelli”, Naples, Italy; ^2^Department of Biology, College of Science and Technology, Temple University, Philadelphia, PA, USA; ^3^Department of Biology, University of Naples “Federico II”, Naples, Italy; ^4^Department of Chemical Sciences, University of Napoli Federico II, Napoli, Italy; ^5^Department of Basic Medical Sciences, Neurosciences and Sense Organs, University of Bari “Aldo Moro”, Bari, Italy; ^6^Department of Interdisciplinary Medicine, University of Bari “Aldo Moro”, Bari, Italy; ^7^Department of Biosciences, Biotechnologies and Biopharmaceutics, University of Bari “Aldo Moro”, Bari, Italy

## Abstract

Beer is one of the most consumed alcoholic beverages in the world, rich in chemical compounds of natural origin with high nutritional and biological value. It is made up of water, barley malt, hops, and yeast. The main nutrients are carbohydrates, amino acids, minerals, vitamins, and other compounds such as polyphenols which are responsible for the many health benefits associated with this consumption of drinks. Hops and malt are one of the raw materials for beer and are a source of phenolic compounds. In fact, about 30% of the polyphenols in beer comes from hops and 70%-80% from malt. Natural compounds of foods or plants exert an important antioxidant activity, counteracting the formation of harmful free radicals. In the presence of an intense stressing event, cells activate specific responses to counteract cell death or senescence which is known to act as a key-task in the onset of age-related pathologies and in the loss of tissue homeostasis. Many studies have shown positive effects of natural compounds as beer polyphenols on biological systems. The main aims of our research were to determine the polyphenolic profile of three fractions, coming from stages of beer production, the mashing process (must), the filtration process (prehopping solution), and the boiling process with the addition of hops (posthopping solution), and to evaluate the effects of these fractions on Dental-derived Stem Cells (D-dSCs) and human intestinal epithelial lines (Caco-2 cells). Furthermore, we underline the bioavailability of beer fraction polyphenols by carrying out the *in vitro* intestinal absorption using the Caco-2 cell model. We found an antioxidant, proliferating, and antisenescent effects of the fractions deriving from the brewing process on D-dSCs and Caco-2 cells. Finally, our results demonstrated that the bioavailability of polyphenols is greater in beer than in the control standards used, supporting the future clinical application of these compounds as potential therapeutic tools in precision and translational medicine.

## 1. Introduction

Polyphenols are organic compounds with one or more hydroxyl groups attached to one or more phenyl rings, they have a protective role in plants, and they are thus present in our diet in plant-based food [1, 2]. The power of polyphenolic compounds in the reduction of oxidative processes is related to their reactive specie (RS) scavenging activity. The pathogenesis of many age-associated diseases can be due to an increase in oxidative stress. In the biological system, the most common source of free radicals is oxygen while the most abundant source of free radical formation is the mitochondria which use more than 90% of the oxygen supply to burn proteins, lipids, and hydrocarbons and convert them into energy and water. On this basis, the high-calorie theory of aging and the theory of free radicals on aging and related senescence [3] were born. The overproduction of reactive oxygen and nitrogen species (ROS/RNS) causes oxidative damage to cellular components, including lipids, proteins, and nucleic acids, predisposing to age-related disorders. Thus, ROS/RNS causes cell dysfunction and physiological decline, leading to aging, with the appearance of degenerative diseases, and eventually death. Aging is characterized by a progressive deterioration, which leads to a loss of functionality of the cells, tissues, organs, and finally to death; it is an irreversible process affecting all higher organisms. Cellular senescence is a process that can be accelerated by using nonlethal stress. This phenomenon is referred to as premature stress-induced senescence (SIPS). Premature senescence of cultured cells is generally associated with environmental stress factors. Different genotoxic agents, such as hydrogen peroxide (H_2_O_2_), ethanol, mitomycin C, tert-butyl hydroperoxide, copper sulphate, thermal shock, and UV radiation, are well-established inducers of SIPS. In toxicology, SIPS can be used to identify xenobiotics inducing premature senescence. However, oxidative stress is the main cause of SIPS activation in normal cells [3, 4].

The antioxidants that manage to eliminate ROS/RNS are able to slow down the aging and related senescence process [4]. Different plant-derived antioxidants, in particular polyphenols, may have a therapeutic potential for aging and age-related diseases. Several studies have shown that polyphenols prolonged the life span of several species. These studies have been carried out in the yeast and then confirmed also in other species, such as *Caenorhabditis elegans*, *Drosophila melanogaster*, and mice. In particular, yeast cells have been a good model for assessing the antioxidant capacity of polyphenols during cellular oxidative stress, mainly because the defense mechanisms and adaptation to oxidative stress are well established and can be extrapolated to human cells [5].

Hydroxyl groups (OH) on ring structures of phenolic compounds make them capable of capturing free radicals (highly reactive oxygen species) and blocking the spare oxygen for other reactions [6]. In particular, polyphenols exert their natural antioxidant action by scavenge-free radical by H-atom transfer from the active OH group(s) of the polyphenolic to the free radical. When they are involved in such reactions, they oxidize themselves to quinones, by donating hydrogens of the hydroxyl groups on the phenolic ring. Antioxidant can also act by chelating metals like iron, copper, and manganese that catalyse the formation of radicals. In fact, they promote the decomposition of H_2_O_2_ in hydroxyl radicals, which are very powerful oxidants, capable of initiating radical chain reactions by extracting hydrogen from almost any molecule [2, 7]. Dietary polyphenols play an important role in human health. Polyphenols are present in many edible plants for both men and animals, and it is believed that their presence, along with that of other molecules such as carotenoids, vitamin C, or vitamin E, is responsible for the health effects of fruit and vegetables [8–11]. In addition, polyphenols are bioactive compounds useful in preventing chronic disorders that may achieve large concentrations in the gut tract, which may be important in the context of intestinal and Autism Spectrum Disorders (ASD) by “gut-brain axis” [12, 13]. A high intake of foods rich in polyphenols has been associated with the improvement of the wellbeing and a decrease in the risk of many diseases including inflammation, cardiovascular, degenerative diseases, and cancer, as well as all health conditions related to plasma proteomic biomarkers [14–16]. The bacterial species of the oral cavity are manifold and contribute to the development of various oral pathological conditions such as dysbiosis, infections, caries, dysplasia, periodontitis, as well as being involved in immunological disorders, and proliferative diseases [15, 16]. Many studies have reported that natural compounds such as polyphenols have inhibitory effects on some oral pathogenic microorganisms [7]. The health effects of polyphenols are dependent on their bioaccessibility and bioavailability. Polyphenols are able to interact with proteins through hydrogen bonds or hydrophobic interactions, forming soluble or insoluble aggregates. These complexes can influence the antioxidant activity of polyphenols and their bioavailability [17]. According to their different chemical properties, polyphenols may be divided into 4 different categories: flavonoids, stilbenoids, phenolic acids, and lignan. In most cases, the food contains a complex mixture of polyphenols, whilst some polyphenols are specific components of certain foods, for example, flavanones in citrus fruits, isoflavones in soybeans, and phloridzin in apples [6]. Other sources of polyphenols are red wine, rich in resveratrol, extra virgin olive oil, rich in hydroxytyrosol, dark chocolate, and tea, especially green tea, rich in epigallocatechin gallate (EGCG), and beer, rich in polyphenols such as Naringenin, Catechin, Quercetin, Rutin, Arbutin, and alkaloids such as Berberine. Naringenin is considered to have a bioactive effect on human health as an antioxidant, free radical scavenger, anti-inflammatory, etc. [6]. Catechin is present in many fruits such as apples, blueberries, kiwi, strawberries, green tea, red wine, beer, cacao liquor, chocolate, and cocoa with an antioxidant action well-established by various scientific evidences using *in vitro* and *in vivo* methods [2, 17]. Moreover, it seems to play a role in molecular mechanisms involved in angiogenesis, extracellular matrix degradation, cell death, etc., thanks to its antioxidative, antihypertensive, anti-inflammatory, antiproliferative, antithrombogenic, and antihyperlipidaemic actions. Quercetin shows a potential chemo-preventive activity; it may produce antiproliferative effects and also produce anti-inflammatory and antiallergy effects mediated through the inhibition of the lipoxygenase and cyclooxygenase pathways, thereby preventing the production of proinflammatory mediators [7]. Rutin is able to chelate the bivalent iron contained in haemoglobin, preventing radical reactions with hydroxyl and peroxyl ions. It therefore acts as an antioxidant and strengthens the capillary wall reducing bleeding symptoms such as those related to hematomas or haemorrhoids; it can reduce the oxidation effects of LDL cholesterol, decrease the risk of heart disease, and also possess antihistamine activity [14]. Arbutin is considered a prodrug, inactive in the glycosylated form but active after being metabolised in hydroquinone, having a disinfecting action in the urinary tract [17]. Berberine is a substance with a bitter taste and an intense yellow colour, traditionally used to treat infections of various kinds thanks to its antimicrobial and ant secretive properties. In the last years, interest has also been linked to its hypocholesterolemic and hypoglycaemic properties, as it is considered a possible natural alternative to statins [18]. Beer is one of the oldest and most widely consumed drinks in the world and the third most popular drink overall after water and tea [19]. It is a beverage that, equally to wine, has a significant energy content and is rich in chemical compounds of natural origin with high nutritional and biological value. It is made up of water, barley malt, hops, and yeast. The main nutrients are carbohydrates, amino acids, minerals, vitamins, and other compounds such as polyphenols which are responsible for the many health benefits associated with this consumption of drinks [18]. Hops and malt are one of the raw materials for beer and are a source of phenolic compounds. In fact, about 30% of the polyphenols in beer come from hops and 70%-80% from malt [20]. The main antioxidant compounds in beer are phenolic compounds and melanoidins (formed throughout Maillard reaction). In addition, some antioxidant additives used in beer (i.e., vitamin C) may also contribute to its antioxidant capacity. Moreover, hops provide a resin containing monoacyl phloroglucinols becoming bitter acids during the development process of beer, such as *α*-acids (humulones) and iso-*α* acids. The structural classes of polyphenols in beer include simple phenols, benzoic acid derivatives, and cinnamic acid, coumarins, catechins, di- and trioligomeric proanthocyanidins, prenylated chalcones, and *α*- and iso-*α* acids derived from hops [21].

Total polyphenols and phenolic acid contents greatly vary among different beer types. In various commercial beers types (abbey, ale, bock, wheat, lager, pilsner, and dealcoholized), ferulic acid is the most abundant phenolic acid in beers, followed by synaptic, vanillic, caffeic, p-coumaric, and 4-hydroxyphenylacetic acids. Ferulic, caffeic, syringic, synaptic, and vanillic acids are present in beers mainly as bound forms, whereas p-coumaric and 4-hydroxyphenylacetic acids are generally present equally in free and bound forms [22–24].

In malt, there are different enzymes, each with different actions, which operate optimally at different temperatures and acidity (pH) [24]. The most important are diastasis, which degrades the starches of the cereal into sugars. In the mashing phase, the ground cereal is mixed with hot water to allow the activation of the enzymes contained in the malt forming, in this way, the must solution. These require particular temperature (and acidity) conditions. The water/cereal mixture is gradually brought to certain temperature levels by direct heating of the dough [7].

Once the action of the diastase enzymes has been verified, i.e., after the starches have been completely transformed into sugars, it is necessary to filter the dough. Usually, this phase is carried out with the help of a double bottom which, by supporting the threshes, allows the sugar must to be filtered until it is free of impurities [20]. For a more effective action, the first must (more turbid) is brought back to the filter vat to undergo further filtration (prehopping solution) [21].

The boiling of the must after filtration is normally carried out for 60-90 minutes performing various functions, i.e., favour the coagulation and precipitation of proteins and polyphenols. At the beginning of the biling phase, hops were added. From an organoleptic point of view, hops have both a bittering function, given by the alpha acid component, and an aromatic one, given by beta acids and essential oils. As mentioned, the bittering function may be only possible through the solubilization of the alpha acids of the hops by means of prolonged boiling. The must after boiling, however, is low in oxygen, essential for proper fermentation [20]. The brewer then replenishes the amount of oxygen needed through various methods, such as blowing in the must of pure oxygen or sterile air or simply with mechanical aeration (i.e., by dropping the must into the fermenter from a certain height or simply stirring the must in the fermenter), forming the posthopping solution. The must is then ready for the addition of yeast and the fermentation phase [24].

Senescence has a dualistic function: from one side, it protects cells from damage and oncogene activation but on the other, it limits the possibility for tissue regeneration triggering aging-related deterioration. In a sense, senescence is the recapitulation, in the cell, of aging in an organism [25]. In the last decades, the use of synthetic and natural molecules has contributed to the development of strategies that may counteract stem cell senescence allowing for the preservation of tissue homeostasis and our innate self-healing potential. On the other hand, although numerous studies have been conducted and many promising results have been reported, investigating new antisenescence strategies to achieve effective stem cell-based therapy remains crucial.

Mesenchymal stem cells (MSCs), adult stem cells with the potential of differentiation into different lineages, play an important role in tissue homeostasis and regeneration [26, 27]. Data show that ROS increase in aged MSCs, and accumulated oxidative damage leads to abnormal proliferation and ultimately MSC senescence [25, 28].

MSC senescence is a complex and comprehensive problem, so multiple different approaches are required to counteract or prevent senescence and improve the clinical application of MSCs.

Polyphenols and other natural molecules could improve different properties (i.e., osteogenic) in MSCs such as Dental-derived Stem Cells (D-dSCs) [29, 30]. Importantly, these cells are easily collected from surgically removed tooth, thus allowing for the reuse of biological waste. The highly proliferative and self-renewing population of dental stem cells has the neural crest as their origin. This expands their applicability for the regeneration of tissues from both ectomesenchyme and mesenchymal origins [31]. Ease of tissue harvest, high initial yield of cells, low population-doubling time, plasticity, multipotential capabilities, and immunomodulatory properties make them a suitable candidate for various therapeutic strategies [27].

Many studies have shown positive effects of beer compounds on biological systems. In particular, antioxidant and anti-inflammatory activities have been described in enzymatic experiments and *in vitro* cells [23]. We have previously demonstrated the presence of bioactive compounds in the wastewater during beer production and evaluated the efficacy of isolated phenolic compounds in SH-SY5Y tumor cells and through the Folin-Ciocalteu assay; it was found that 2.30% was made up of phenolic compounds. In addition, tumor cells treated with the polyphenolic extract showed a decrease in oxidative stress and an increase in mitochondrial biogenesis [23].

The first objective of this study was to investigate about a qualitative and quantitative analysis of the polyphenols content in three fractions derived from the mashing process (must), the filtration process (prehopping solution), and the boiling process with the addition of hops (posthopping solution).

To our knowledge, there are no studies evaluating the effect of beer polyphenols on D-dSCs. An important second aim in this translational research is to study the antioxidant capacity and antisenescent effect of three fractions, must, prehopping solution, and posthopping solution on D-dSCs and human intestinal epithelial lines (Caco-2 cells). Furthermore, as a predictor of bioavailability of beer polyphenols, we have carried out the intestinal absorption using the human Caco-2 cell line model.

## 2. Material and Methods

### 2.1. Chemicals and Standards

Naringenin, Catechin, Quercetin, Rutin, Arbutin, and Berberine (99%) were purchased from Sigma Aldrich (Milano, Italy). These polyphenols will serve as standards in high-pressure liquid chromatography (HPLC) analysis. All the other chemicals and solvents were purchased from Fluka (Saint-Quentin Fallavier, France) with HPLC grade and were used as received.

### 2.2. Beer Process Samples

Brewing fractions provided from the mashing process (must), the filtration process (prehopping solution), and the boiling process with the addition of hops (post-hopping solution) of the beer called “*La Meridionale*” of the Birrificio Bari (Italy), a blanche quality (Master Brewer: Paola Sorrentino) made with the addition of Gargano's IGP oranges, a spice coriander, and borage, a wild plant typical of the Mediterranean maquis, were used for the experimental procedures. The three fractions considered were first filtered and then subjected to HPLC analysis [32] for their content in polyphenols.

### 2.3. HPLC Analysis

HPLC was performed on a Shimadzu LC-8A by using Shimadzu SPD-10A VP UV-VIS detector (Shimadzu, Milan, Italy). The reverse-phase HPLC analyses were performed using an Agilent Eclipse XDB C_18_ analytical column (5 *μ*m particle size, 150 mm × 4.6 mm i.d.), under isocratic conditions, using an eluent mixture of methanol, and water containing 0.2% of acetic acid (50 : 50, *v*/*v*), at 25°C. The flow rate was 0.7 mL/min, injections were 20 *μ*L in volume, and the detection wavelength was 280 nm. A standard stock solution (100 mg/L) of Arbutin was prepared in methanol. Aliquots of this stock solution were diluted to different concentrations in the calibration range 0.2–20 mg/L. Grade chemicals were procured from Merk (Germany).

The calibration curve was constructed employing dependence between peak areas and concentration, using the mean values of three determinations for each concentration. Spectrophotometric detection at 280 nm was carried out. The calibration curve for all other flavonoids was prepared at the same chromatographic conditions as Arbutin. Commercial stock components of Naringenin, Catechin, Quercetin, Rutin, Arbutin, and Berberine, taken by Sigma Aldrich (USA), served as standard in the construction of the calibration curves using seven solutions with a concentration ranging between 0.1 and 500 ppm.

### 2.4. In Vitro Cell Culture Studies

Dental-derived Stem Cells (D-dSCs) have been obtained from apical papilla tissue that surrounds the developing tooth of healthy donors and cultured *in vitro*, as previously described [33, 34]. All patient's parents and guardians gave permission after they signed a written informed consent in accordance with the Helsinki Declaration, for the reuse of human biospecimens in scientific research. The investigation was conducted in accordance with the current medical protocol as described by the Italian Government's NIH legislation. Procedures were performed following our previous experience in the field and according to manufacture specifications. Cells were cultured under standard conditions as described [34]: 95% air and 5% CO2 at 37°C, to retain the proliferative condition, and grown, respectively, in RPMI medium containing 10% fetal bovine serum (FBS), 100 IU/mL penicillin, and 100 *μ*g/mL streptomycin. Cells were collected, and quantification was performed using a hemocytometer chamber in order to seed an equal number of viable cells for each experiment. The cells were seeded at a final density of 10,000 cells/well and in 6-well culture plates. The in vitro experiments were performed in triplicate to ensure that the results would be reproducible.

Human intestinal epithelial lines (Caco-2 cells) were obtained from ATCC (Philadelphia, PA, USA). They were cultured in DMEM supplemented with 10% fetal bovine serum, 1% L-Glutamine, and 1% penicillin/streptomycin. Cells were cultured under standard conditions in a humidified atmosphere containing 95% air and 5% CO2 at 37°C. The cells used in the experiments were between passages 20 and 30. Cells in log-phase growth were subcultured weekly by trypsinization and were seeded at ratio 1 : 3 upon reaching 80% confluence, as described in [35].

### 2.5. Cell Proliferation Assay

Cell viability assay was performed by the MTT [3-(4,5-dimethylthiazolyl)-2,5-diphenyl tetrazolium bromide] assay as described in [36, 37]. Briefly, cells 3 × 10^3^ were seeded in 96-well plates and pretreated with three fractions, must, prehopping solution, and posthopping solution, coming from stages of beer production for 24 h. We tested 25, 50, and 100 *μ*L of each beer fractions. Untreated cells (Ctr) were used as negative control. Another MTT assay was performed under oxidative stress conditions: cells were pretreated with the beer fractions as above and then they were subjected to oxidative stress with H_2_O_2_ 180 *μ*M for 30′ at 37°C. Cells treated with H_2_O_2_ 180 *μ*M for 30′ at 37°C without pretreatment with beer fractions were used as positive control. MTT solution at 10% was added to each well and incubated for 2 h. Then, excess medium was removed, and 100 *μ*L of a solution of 1 N hydrochloric acid at 10% in isopropanol was added to dissolve the formazan crystals. The mixture was shaken for about 20 min, and the optical density in each well was measured using a microplate spectrophotometer (Microplate Reader Model 550, Bio-Rad, California, USA) at 570 nm. Triplicate experiments were performed for each condition [38, 39]. The cell viability percentage (%) was calculated by comparison with a sample's corresponding control.

### 2.6. Transepithelial Transport Studies

For the transport experiments [40, 41], the cells were seeded at a density of 5 × 10^5^ cells/cm^2^ in 6-well filter support inserts with polyethylene ether phthalate membranes (0.4 *μ*m pore size, 23.1 mm diameter, 4.2 cm^2^ growth surface area (BD Falcon, Italy). The medium was changed every alternate day till usage. The cells were differentiated for at least 14 days prior to the transepithelial transport experiments. The integrity of the cell layers was evaluated by transepithelial electrical resistance (TEER) measurements using an epithelial Volt-Ohm Meter (Millicell ERS-2, Millipore, Italy). Only the Caco-2 monolayers with TEER values higher than 400 *Ω*/cm^2^ were selected for the subsequent experiments. Following the TEER value measurement, the Caco-2 cell monolayers were gently rinsed twice with PBS, and transport medium (HBSS supplemented with 25 Mm glucose and 10 Mm HEPES, TM) was added to the apical (2 mL) and basolateral (3 mL) compartments. Following a 240′ incubation at 37°C, the solution from the apical and basolateral compartments was collected for HPLC analysis.

### 2.7. Senescence-Associated *β*-Galactosidase Staining

The senescence marker, senescence-associated beta-galactosidase (SA-*β*-Gal), was detected as previously described [42]. Briefly, cells were cultured up to about 80% confluence; before the experiments, cells were seeded onto 24-well plates at a density of 10 × 10^3^ cells/cm^2^. After the time to allow the cell attachment (24 h), the medium was replaced in each well and the cells received H_2_O_2_ 180 *μ*M for 30′ at 37°C. Subsequently, after H_2_O_2_ senescence induction, the three fractions (must, prehopping, and posthopping solutions) were added and maintained in the medium for three days before the SA-*β*-Gal staining assay. In parallel experiments, the three fractions of beer production were added to the cell medium and maintained for three days before assessing the SA-*β*-Gal staining. The number of senescence-associated SA-*β*-Gal-positive cells was determined in 100 randomly chosen low-power fields (X 100) and expressed as a percentage of all counted cells.

### 2.8. Statistical Analysis

Experiments were executed in three replicates, and the results were expressed as the average ± standard deviation (SD).

The in vitro antisenescence activity data are presented as the means ± standard errors of the means (SEM) of triplicate samples and are the representative of three different experiments. Statistical analysis was performed by one-way analysis of variance (ANOVA) with Bonferroni's corrected *t-*test. A difference between means was considered significant if *p* ≤ 0.05.

## 3. Results

### 3.1. HPLC Analysis of Beer Fractions

**Figure 1:**
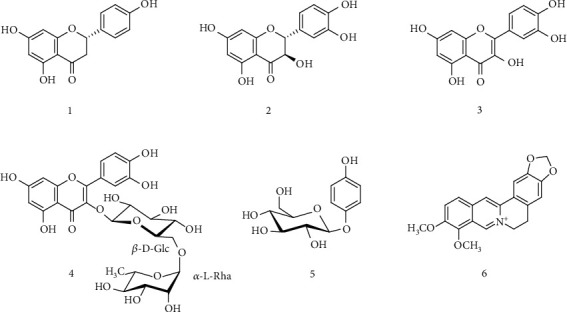
Structure of quantified compounds: (1) Naringenin, (2) Catechin, (3) Quercetin, (4) Rutin, (5) Arbutin, and (6) Berberine.

The phenolic compounds in beer are related to the composition of the raw materials used in the brewing process. In addition to the genetic characteristics, the fermentation phase may also interfere with the final concentration of polyphenols. Therefore, it is important to evaluate the polyphenol content in fractions coming from different stages of beer production. In particular, we have performed a quantitative and qualitative analysis of the polyphenol content in three fractions, must, prehopping solution, and posthopping solution. The presence of one flavanone as Naringenin (1), one flavan-3-ol as Catechin (2), two flavanols as Quercetin (3), and Rutin (4), one glycosylated hydroquinone as Arbutin (5), and one benzyl-isoquinoline alkaloid as Berberine (6) (Figure 1) have been quantified in the fractions: must, pre-, and posthopping solutions.

The results expressed in ppm are reported in Table 1.

In the posthopping solution, we found an increase in all the measured components. In particular, the flavonoids Rutin (4), Naringenin (1), and Quercetin (3) show a significant increase in the concentration of about eight (27.39 ± 1.62), three (1.96 ± 0.07), and two (0.58 ± 0.04) times higher than the must solution, respectively. The content of Catechin (2) remains almost constant. However, the most abundant component is the glycosylated hydroquinone Arbutin (5) up to a concentration of 352 ppm in the posthopping solution, about 50% compared to its estimated content in the must (243.59 ± 8.31), while the content of the benzyl-isoquinoline alkaloid Berberine (6) is almost the same than in the must solution and quantitatively comparable to flavonoids (1-3).

### 3.2. MTT Assay

**Figure 2:**
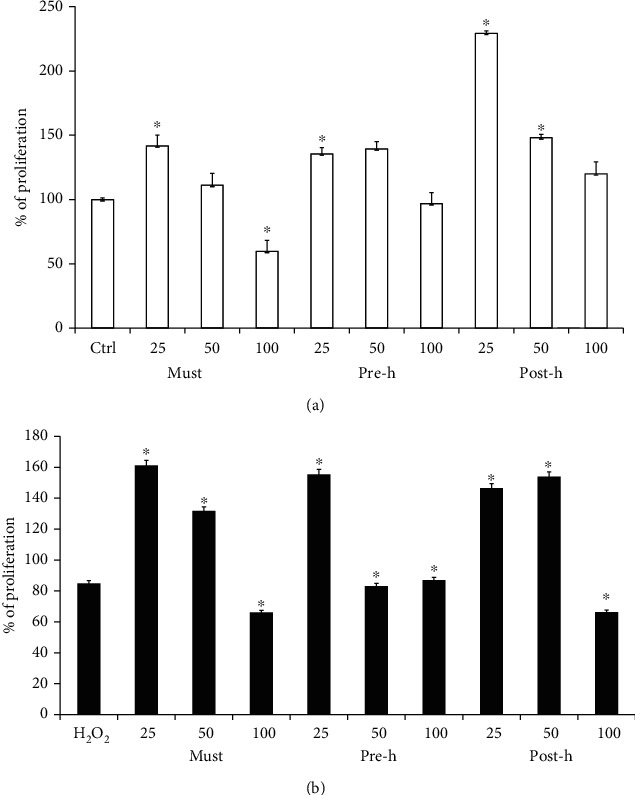
Effect of beer fractions on D-dSCs. D-dSCs viability was determined by MTT assay. (a) Cells treated with beer fractions solution: must (must), prehopping solution (pre-h), and posthopping solution (post-h). (b) Cells pretreated with beer fractions as above and then stimulated with H_2_O_2_ 180 *μ*M for 30′. The histogram shows % of cell proliferation normalized to untreated control cells. The quantity of single treatment is expressed in *μ*L. The statistical analysis derived from at least 3 experiments in triplicate (Student's *t*-test) is shown ^∗^*p* < 0.05 compared to the untreated control.

In order to study the effect of must, prehopping, and posthopping solutions, coming from stages of beer production, on D-dSCs, we have treated the cells for 24 hours with the indicated quantities of solution, and cell proliferation was measured by MTT assay. The corresponding concentration of determinated polyphenols in this tested volume is shown in Table 2. A significant increase of cell proliferation was observed for the cells treated with the fractions at lower concentrations. Contrarily, an antiproliferative effect is observed at higher concentrations of must (100 *μ*L), probably due to a sugar increase in the fraction. High doses (100 *μ*L) of pre- and posthopping solutions did not seem to influence cell growth (Figure 2(a)).

Next, to evaluate the antioxidant effect of polyphenols in the beer fractions, we have pretreated D-dSCs with the three beer fractions as above and then subjected to oxidative stress with H_2_O_2_ 180 *μ*M for 30′ at 37° C. The cell proliferation was measured by MTT assay, and the results showed that the oxidative stress was reverted by the addition of beer samples for 24 hours at the lowest dose (25 *μ*L). This phenomenon was completely abrogated at a high dose (100 *μ*L) of all beer fractions, while the medium doses (50 *μ*L) had different effects depending on the fraction used. In particular, the must and posthopping solution significantly induced an increase in cell proliferation (Figure 2(b)).

**Figure 3:**
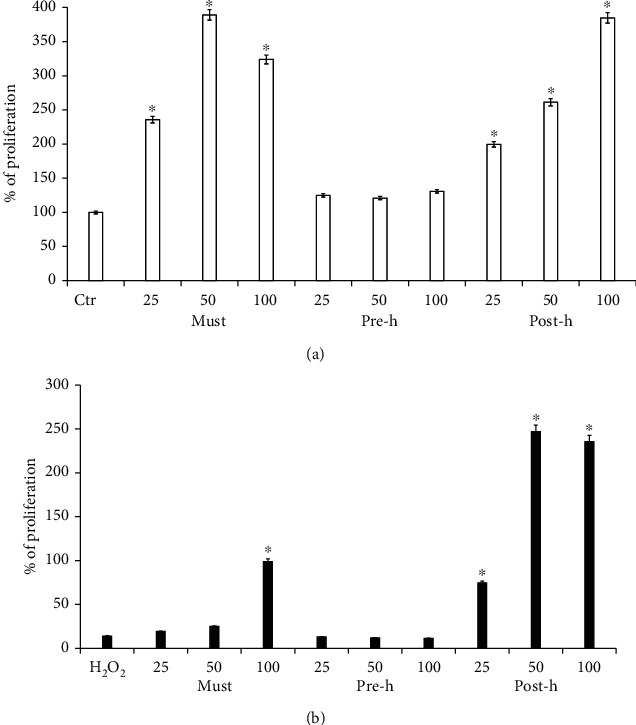
Effect of beer fractions on Caco-2 cells. Caco-2 cell viability was determined by MTT assay. (a) Cells treated with beer fractions solution: must (must), prehopping solution (pre-h), and posthopping solution (post-h). (b) Cells pretreated with beer fractions as above and then stimulated with H_2_O_2_ 180 *μ*M for 30′. The histogram shows % of cell proliferation normalized to untreated control cells. The quantity of single treatment is expressed in *μ*L. The statistical analysis derived from at least 3 experiments in triplicate (Student's *t*-test) are shown ^∗^*p* < 0.05 compared to the untreated control.

The potential intestinal absorption of beer polyphenols was assessed using Caco-2 intestinal cells. Caco-2 cells differentiate into a monolayer of polarized cells, coupled by junctions, which express many morphofunctional features of the absorbent epithelium of the small intestine. Different amounts of beer polyphenols were tested to check the cellular viability using MTT assay before performing the permeability assay. This is to prevent damage to the Caco-2 monolayer.  Figure 3 shows statistically significant cell viability. As shown in Figure 3(a), no concentration tested was toxic, indeed the increasing of cell viability in treated cells compared to controls was observed in must and posthopping solution. Next, we performed the MTT assay under oxidative stress conditions: Caco-2 cells were pretreated with the beer fractions as above, and then they were subjected to oxidative stress with H_2_O_2_ 180 *μ*M for 30′ at 37°C. As shown in Figure 3(b), after oxidative stress, an increase in cell viability was observed when Caco-2 cells were treated with high doses of must and posthopping fractions.

### 3.3. Effects of Beer Fractions in Cellular Senescence

**Figure 4:**
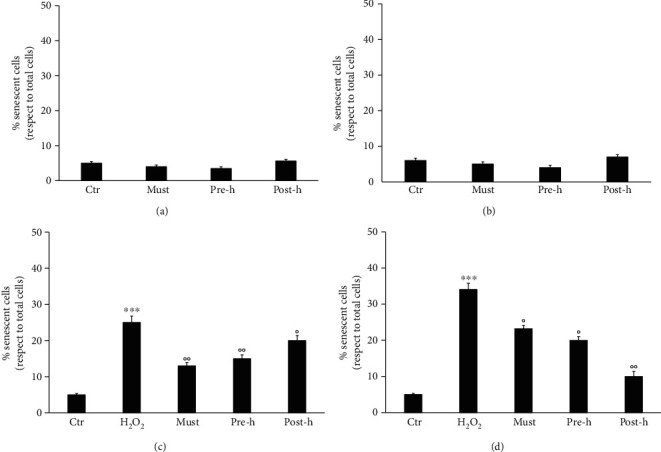
Antisenescent effect of beer fractions on D-dSCs and Caco-2 cells. Senescence-associated beta-galactosidase (SA-*β*-Gal) was detected. (a) D-dSCs; (b) Caco-2, percentage of cellular senescence in not-injured cells treated with beer fractions solution: must (must), prehopping solution (pre-h), and posthopping solution (post-h). (c) D-dSCs; (d) Caco-2, percentage of cellular senescence in H_2_O_2_-injured cells treated with beer fractions solution: must (must), prehopping solution (pre-h), and posthopping solution (post-h). Data is shown as the percentages of *β*-galactosidase-positive cells with respect to the total cell number of the sample. Each bar represents the mean ± SEM of three replicates from three independent experiments. ^∗∗∗^*p* < 0.001 versus the control (Ctr, cells not injured); °°*p* < 0.01 versus the H_2_O_2_; °*p* < 0.05 versus the H_2_O_2_.

To explore the protective potential of beer fractions against cellular senescence, the H_2_O_2_-treated D-dSCs and Caco-2 cells were cultured with fresh complete medium containing the three beer fractions: must, prehopping solution, and posthopping solution, and the pivotal senescence hallmark, the SA-*β*-Gal staining was assessed after 3 days. As reported in Figures 4(a) and 4(b), beer fractions alone did not affect the number of senescent cells with respect to control, for any of the tested cells. Figures 4(c) and 4(d) showed the results obtained from the H_2_O_2_-induced senescence model. D-dSCs and Caco-2 cells injured with H_2_O_2_ evidenced a significant SA-*β*-Gal staining in respect of control cells, and notably, all the three beer fractions were able to protect the injured cells from the appearing of the senescence hallmark.

### 3.4. Transport Test

**Figure 5:**
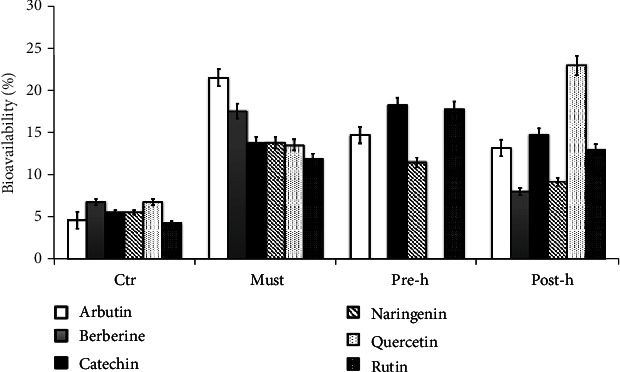
Bioavailability of beer polyphenols in the Caco-2 cell model. Cells were treated with beer fractions: must (must), prehopping solution (pre-h), and posthopping solution (post-h), for 4 h. The bioavailability of individual polyphenols in beer extracts is expressed as % of polyphenols which exceeds the apical compartment and passes into the basolateral compartment and collected for HPLC analysis.

To investigate whether the polyphenols of beer fractions (must, prehopping, and posthopping solutions) were bioavailable, Caco-2 cells in monolayer with TEER values higher than 400 *Ω*/cm^2^ were treated at the apical and basolateral compartments with transport medium (see Material and Methods section) for 240′ at 37° C. Then, solutions from the apical and basolateral compartments were collected for HPLC analysis.  Figure 5 shows the percentage (%) of bioavailability of the main identified polyphenols in culture media. According to transepithelial transport studies, the amount of polyphenolics recovered on the apical side gives some information on polyphenols stability in the culture medium, in relation to the incubation time. Instead, the phenolic concentration recovered in the basolateral side gives time-related information on polyphenols/metabolites transport. Our results show that all polyphenolics recovered were bioavailable more than the 10%, except for Berberine and Naringenin in the posthopping solution and Berberine and Quercetin in the prehopping solution. Arbutin and Berberine compound from must solution, Catechin and Rutin compound from prehopping solution, and Quercetin compound from posthopping solution were transported through the human intestinal epithelium (Caco-2 cells) with a higher intestinal permeability (15-25%) (Figure 5).

It is interesting to note that the standards used have a lower permeability than the same polyphenols present in the beer fractions; this is probably due to the fact that in their natural source there are interactions and synergies that are not predictable and replicable *in vitro*.

## 4. Discussion

The ROS/RNS produced by several endogenous and exogenous processes is neutralized by cellular antioxidant defenses. Imbalance between ROS/RNS and the antioxidant defenses results in cellular oxidative stress [3, 25]. This imbalance occurs in the case of aging and leads to progressive loss of tissue and organ function. Stem cell senescence and functional decline have emerged among the core features of aging, underlying deficient regenerative potential, and lost tissue homeostasis in various organs/systems [25, 28]. Natural compounds are commonly used for the prevention of various senescence changes, such as antioxidants and anti-inflammatory agents [23]. So, naturally occurring compounds polyphenols are secondary metabolites of plants, comprised of several categories, namely, flavonoids, phenolic acids, lignans, and stilbenes. In recent years, the potential antisenescence benefits of polyphenols have been gaining increasing scientific interest due to their capability to modulate oxidative damage, inflammation, and autophagy [23, 29]. Numerous cohort studies have pointed out that consumers of light to moderate alcohol have greater survival than abstainers [43–46]. In particular, the positive effects are on cellular senescence damage, cognitive function, and dementia. These effects have been observed in a variety of patients, including diabetics and hypertensive subjects. A part of these effects may be certainly attributed to the polyphenols contained in beer and wine, since these compounds have antioxidant properties, anti-inflammatory, anticarcenogenic, hypotensive, or even anticoagulants [2, 47] and should be considered a useful tool in controlling cell senescence.

Current understanding of polyphenols impacts on adult stem cells mainly comes from *in vivo* conditions of tissue injury and regeneration as well as *in vitro* situations of proliferation and induced differentiation. Polyphenols are found mainly in the peripheral layers of the malt bound to the polysaccharides of the cell wall, in quantities higher than the corresponding varieties of barley. During malting, there is a significant increase in phenolic compound levels thanks to enzymatic processes. Barley polyphenols are very important due to their influence in the different stages of the fermentation process and the overall stability of the beer [43]. However, the excessive presence of polyphenols is considered a problem for craft beer, so every process tends to reduce its content to avoid harsh flavors and excessive turbidity when this is not required by the desired style. There are many moments in which it is possible to analyse the beer in its production phases, to be able to test and quantify the presence of polyphenols to evaluate the beneficial effects on humans.

In the current study, fractions deriving from the brewing process were investigated for the evaluation of polyphenols content, antioxidant and proliferating action, antisenescent properties, and bioavailability *in vitro*. Our analysis about the chemical composition of beer fractions led to the identification and quantification of six polyphenols (Naringenin, Catechin, Quercetin, Rutin, Arbutin, and Berberine) from must, pre-, and posthopping solutions during beer production. The results of this study show an increase in posthopping solution of Rutin and Arbutin of about eight and two times, respectively, compared to the must, while Naringenin increases its concentration by about three times compared to the must. Hydroquinone Arbutin is the most concentrated polyphenol in the posthopping solution. Rutin has significant anti-inflammatory and immunomodulatory effects and is a bioactive compound in pharmaceutical products with the action of free radical scavenger [48]. Naringenin is a flavanone, aglycone of Naringin with various pharmacological activities. In particular, it is used not only in situations of oxidative stress but also in various pathological conditions such as inflammation, cancer, diabetes, cardiovascular diseases, and neurological disorders [49]. Arbutin has an antioxidant, antimicrobial, and anti-inflammatory action. It is used for the treatment of kidney stones, cystitis, and urinary tract infections [50]. Moreover, thanks to the inhibitory effect of tyrosinase, it is used in the cosmetic field, as a powerful skin lightening agent [51]. Furthermore, several studies show that the consumption of polyphenols is also important in dental health. They have different actions such as an inhibitory effect on cariogenic bacteria and bacteria associated with periodontal disease, halitosis, and so on [52–55]. Next, we evaluated the effect of beer fractions on cell proliferation and its antioxidant capacities in two cell systems, D-dSCs, and Caco-2 cells. Cell proliferation was assessed by using MTT assay. This method provides tangible and numerical evidence of cell viability as a function of mitochondrial activity and therefore furnishes a measurement of cell proliferation. Our results indicate that low concentrations of beer fractions induce a significant increase in cell proliferation while at higher doses an antiproliferative action is observed. This could be due to the higher quantity of sugar present in the beer fractions as they are not yet fermented. Beer is known as a source of natural antioxidants that counteract free radicals and reduce senescence. So we evaluated whether there was a correlation between polyphenols and antioxidant properties of beer fractions. In fact, H_2_O_2_ is naturally produced in the human cell and is widely used as a prooxidant model in the study of oxidative stress. Thus, we tested the antioxidant capacity of the beer fractions by inducing oxidative stress with H_2_O_2_. The results reported here indicate that the pretreatment with beer fractions have an antioxidant effect as fractions reverted the oxidative stress induced by H_2_O_2_ in D-dSCs and Caco-2 cells.

Several compounds have recently been identified that can significantly reverse senescence induced by oxidative stress. Moreover, D-dSCs are considered as a useful model in this translational research area also for their stemness properties [56–60]. To evaluate the antisenescence effect of the beer fractions, the D-dSC and Caco-2 cells were exposed to H_2_O_2_ with and without beer fractions and analysed by using *β*-galactosidase assay. It is based on the expression of the enzyme *β*-galactosidase, which is overexpressed in cells in the senescence phase and is defined as SA-*β*-Gal (senescence-associated *β*-galactosidase). As expected, exposure to H_2_O_2_ resulted in the increase in senescence-associated *β*-galactosidase activity in both cell models. The addition of must, prehopping solution, and posthopping solution, coming from stages of beer production to the two cellular systems, significantly reduced the senescence induced by oxidative stress.

The number of reports on the beneficial health effects of polyphenols is increasing speedily but there is still little knowledge about metabolism and bioavailability in humans.

Even if some explanation of bioavailability has been suggested, the better appropriate seems to be as that fraction of an ingested nutrient or compound that reaches the systemic circulation and the specific sites where it can exert its biological action [61–64].

The host-related factors affecting the bioavailability can be further subdivided into intestinal factors and systemic factors. The intestinal factors probably represent the most important ones. This activity is of great importance for the biological action of polyphenols, since active metabolites are produced by the colonic microflora, taking into account the great interindividual variability in producing these active metabolites [62].

The absorption of a substance is to be considered as a limiting factor to the biokinetics of the molecule, and its evaluation is therefore fundamental to define the subsequent toxicological studies. The intestinal absorption model based on Caco-2 cells is a well-characterized *in vitro* system that makes it possible to study the mucosal toxicity of all those substances that are intentionally ingested or accidentally, define their transport mechanism through the intestinal barrier, and then determine their bioavailability in the blood and tissues [65, 66].

Indeed, prior to passage into the blood, the polyphenols undergo former structural modifications due to the conjugation process that takes place in the intestine [61, 62]. To achieve healthy properties, the polyphenols must be bioavailable, effectively absorbed from the intestine into the circulation, and released to the target tissues. The intestinal epithelium is one of the main barriers between the external environment and internal organs. It constitutes a double target of any toxic insults coming from drugs or beneficial bioavailability substances present in the diet.

In the present study, we tested the bioavailability of beer polyphenols using the Caco-2 human intestinal cell model. The relatively high bioavailability values recorded for almost everyone polyphenols in beer fractions have shown that these compounds can be transported through the Caco-2 monolayer with an intestinal permeability higher than that shown by the relative standards used as control. Often, the benefit of some natural products cannot always be explained with the individual components; there are synergies between the various natural constituents which are more effective than the sum of the individual parts. On the other hand, so far, no one has shown that the health effects of some nutrients can be reproduced by isolating their individual components.

Although all these studies have yielded important data for understanding the effects of phenolic beer compounds on cellular systems, much remains to be studied. Further investigation are needed to understand the molecular mechanisms and furthermore human studies as well as experimental animal models are required for a better understanding of the effects of beer, in particular when consumed regularly at moderate doses.

## 5. Conclusion

In conclusion, polyphenols are very stable molecules and can be found in various concentrations in the different fractions coming from the beer production phases, the mashing process (must), the filtration process (prehopping solution), and the boiling process with the addition of hops (posthopping solution). Our translational results showed a variable polyphenolic content in the analysed beer fractions; moreover, they showed a proliferative effect at low concentrations and an antioxidant effect in two different cell systems, D-dSCs and Caco-2 cells. In addition, the three beer fractions used revealed an antisenescent action. Caco-2 bioavailability assays allowed to record good bioavailability values for all the beer fractions analysed, in particular, the results showed that the bioavailability of polyphenols is greater in beer than the control standards used confirming the synergy existing in nature. Currently, natural compounds as beer as well as several other drinks and foods are considered not only nutritious but also potentially useful bio-based products that can be used to prevent various chronic diseases. Therefore, we can speculate that the use of natural molecules should be able to counteract oxidative stress and cell senescence, supporting the future clinical application of these compounds as potential therapeutic tools for age-related degenerative diseases. However, the mechanisms have yet to be fully characterized. Therefore, further studies are needed to fully understand the role of beer polyphenols in determining conclusive evidence on impacts on human health.

## Figures and Tables

**Table 1:** Values are expressed in ppm means from three independent samplings ± SD.

	Must	Prehopping solution	Posthopping solution
Naringenin (1)	0.73 ± 0.02	1.27 ± 0.03	1.96 ± 0.07
Catechin (2)	1.39 ± 0.04	4.26 ± 0.22	1.78 ± 0.05
Quercetin (3)	0.32 ± 0.03	Trace	0.58 ± 0.04
Rutin (4)	3.13 ± 0.22	3.53 ± 0.53	27.39 ± 1.62
Arbutin (5)	243.59 ± 8.31	52.44 ± 2.51	351.94 ± 12.66
Berberine (6)	1.37 ± 0.05	Trace	1.45 ± 0.03

**Table 2:** It is shown the corresponding concentration of polyphenols in the tested volume expressed in *μ*g/*μ*L. The calibration curve was constructed employing dependence between peak areas and concentration, using the mean values of three determinations for each concentration.

	Must			Prehop solution			Posthop solution		
	25 *μ*L	50 *μ*L	100 *μ*L	25 *μ*L	50 *μ*L	100 *μ*L	25 *μ*L	50 *μ*L	100 *μ*L
Arbutin	6,0897	12,1794	24,3588	1,31105	2,6221	5,2442	8,798475	17,59695	35,1939
Catechin	0,03465	0,0693	0,1386	0,106575	0,21315	0,4263	0,044375	0,08875	0,1775
Rutin	0,07835	0,1567	0,3134	0,088225	0,17645	0,3529	0,68465	1,3693	2,7386
Berberine	0,034175	0,06835	0,1367	0,000025	0,00005	0,0001	0,036275	0,07255	0,1451
Quercetin	0,007875	0,01575	0,0315	0,00005	0,0001	0,0002	0,0146	0,0292	0,0584
Naringenin	0,018125	0,03625	0,0725	0,031825	0,06365	0,1273	0,04895	0,0979	0,1958

## Data Availability

The experimental data used to support the findings of this study are included within the article.
